# Prosocial priming and bystander effect in an online context

**DOI:** 10.3389/fpsyg.2022.945630

**Published:** 2022-08-12

**Authors:** Costanza Scaffidi Abbate, Raffaella Misuraca, Chiara Vaccaro, Michele Roccella, Luigi Vetri, Silvana Miceli

**Affiliations:** ^1^Department of Psychology, Educational Science and Human Movement, University of Palermo, Palermo, Italy; ^2^Department of Political Science and International Relations, University of Palermo, Palermo, Italy; ^3^Oasi, Research Institute-IRCCS, Troina, Italy

**Keywords:** bystander effect, priming, prosocial behavior, online, help

## Abstract

The present study tested the effect of priming the concept of prosociality on the bystander effect in an online environment. Participants were sent an e-mail requesting a plea for help and randomly assigned to one of four conditions in a 2 (Bystander: 0 vs. 14) × 2 (Priming: present vs. absent) design. The results demonstrated support for the study hypothesis. As expected, the virtual presence of many others significantly reduced e-mail responsiveness except when the request for help is preceded by prosocial priming. Implications of these findings for the literature on the bystander effect and priming are discussed.

## Introduction

Literature on helping behavior has shown that the presence of people can curb helping behavior on the part of those assisting in an emergency due to a diffusion of responsibility effect [Fischer et al. (2011) for a review]. The present study aims to determine whether the diffusion of responsibility occurs when people physically witness an event and in other, less traditional contexts, such as virtual settings. Diffusion of responsibility could then account for the unresponsiveness of those addressees who receive e-mail requests sent simultaneously to multiple people. Further, the study examines whether a prosocial prime in a virtual context may reduce the responsibility diffusion deriving from the presence of bystanders.

Several decades of studies have highlighted many aspects of the bystander effect, revealing that individuals are less likely to assist someone in difficulty when other people are also on the scene because the intervention is inversely correlated to the number of bystanders. This bystander effect has been documented in many well-known experiments (e.g., Darley and Latané, 1968; Latane and Darley, 1968; Darley et al., 1973). However, many other variables influence bystander intervention, including whether or not the situation is an emergency [a danger is posed to a victim or bystanders, or situations in which a villain has infringed upon the rights of others and prompt action is necessary; Latané and Nida (1981)].

Among the various psychological mechanisms that constitute the cornerstones of the bystander effect–including fear of being judged and collective ignorance—Darley and Latané (1968) mainly emphasized the power of the diffusion of responsibility. Indeed, an individual who witnesses a request for help will be ready to intervene if they assume the responsibility to intervene, that is, to decide that at that moment, assisting is their responsibility and not someone else’s. Nevertheless, the assumption of responsibility is conditioned by the number of people present.

The results of observations carried out in the laboratory, starting from the classic experiments of Darley and Latané (1968) up to more recent studies [Garcia et al., 2002; Hussain et al., 2019; Campos-Mercade, 2021; see also Anderson and Misuraca (2017)], indicate that the willingness to help decreases according to the number of bystanders witnessing the emergency. Although they may perceive a conflict between a moral norm directing action and the possible hesitations associated with the intervention, those who find themselves as lone witnesses to a critical circumstance feel a strong urge to get involved and are aware that this pressure falls solely on them. In the one who finds themselves assisting a critical event together with other stranger people, the norm that induces to help the potential victim is attenuated, not only by a typical uncertainty related to the intervention but also by the presence of the others. There may be a diffusion of responsibility, an alleviation of individual responsibility that leads to the assumption that someone else is ready to act or the belief that someone else has already taken action. As a result, the presumed disapproval for not intervening will also spread among bystanders and be less demanding for the individual witness. It seems established that the number of bystanders generates a responsibility-sharing effect in participants (Dovidio et al., 2006).

Research has shown the consequence of responsibility spread in virtual scenarios, such as telematic communication. In an experiment by Blair et al. (2005), 400 college students received a fictitious e-mail from an imaginary college freshman asking for assistance in accessing the database of some electronic periodicals. Students could be informed that others had also received the same e-mail. Specifically, the variable manipulation involved participants in the first group believing that they had received a personal e-mail with themselves as the only recipient, the second group believing that only one other recipient had received the e-mail with the request, and finally, the third and fourth groups believing that 14 and 49 other recipients had received the same message. Results confirmed that participants were less likely to respond when they knew that other students also received the same letter.

## Priming social behavior

Since the 2000s, some studies influenced by the winds of cognitivism have investigated the effect of priming in the bystander effect, and exciting aspects have emerged (Garcia et al., 2002; Marsh and Ambady, 2007; Scaffidi Abbate et al., 2014). A well-established line in social psychological research is that social knowledge can be spontaneously triggered in an individual’s mind when faced with social stimuli. When individuals perceive their surroundings, the social knowledge conveyed by the relevant stimuli in that context can become available to them automatically in memory (Bargh, 2021). It has also been evidenced that activated knowledge can influence people’s perceptions, decisions, goals, and purposes (Ferguson and Bargh, 2004). It has also been verified that social behavior is often generated spontaneously by the simple occurrence of appropriate situational characteristics (Bargh et al., 1996). As Ferguson and Bargh claim:

“Just as a stereotype presumably becomes associated with a group after repeated group-stereotype pairings, a behavior that a person repeatedly performs in a particular situation or response to a specific another person might become associated in memory with the features of that situation or person. In both cases, the mere perception of the group member or situation might automatically activate the respective stereotype or behavior” (Ferguson and Bargh, 2004, p. 34).

Numerous interpretations have been discussed in favor of this view [i.e., the ideomotor principle, the effects of imitation, and the concept of the schema; for a review, see Dijksterhuis and Bargh (2001)]. Bargh et al. (1996) demonstrated that priming the concept of elder induced behavior associated with, for example, walking more slowly, although people were not conscious of the prime.

It seems reasonable to expect that even prosocial concepts may be activated through priming. Considerable research shows that primes associated with prosociality construct enhance helping behavior. For example, Nelson and Norton (2005) noted that participants subjected to prosocial priming would commit to helping behavior in the future more than participants subjected to neutral priming. Some authors have successfully utilized priming to examine the influence of religious stimuli on prosocial conduct (Pichon et al., 2007; Batara et al., 2016; Scaffidi et al., 2016). Over and Carpenter (2009) verified similar findings in children.

The relationship between priming and the bystander effect has been considered in this arena. The aim was to investigate whether the priming of prosocial constructs influences the probability of bystander intervention. Garcia et al. (2002) combined the priming paradigm with the bystander effect literature. They showed that priming people with a social context in which there was only one person (vs. a group of individuals) influences helping behavior on a later unconnected task. Abbate et al. (2013) tested priming in the setting of the bystander arena showing prosocial primes were more likely to prompt helping behavior than neutral primes. The impact of priming also continues in the presence of bystanders. Finally, Meier et al. (2021) observed the effect of priming the construct of responsibility in a field experiment.

This research aims to examine whether activating concepts related to the construct of prosociality can reduce bystander apathy in an online context. Thus, we are interested in understanding two points. First, we test whether the bystander effect occurs even in online contexts, as Blair et al. (2005) found. In the case of a positive answer, we aim to assess whether the priming could reduce the bystander effect. The rationale is that in the case in which a request for help made to a single person is more likely to be considered than a request made when several people are present together–because of the concept of diffusion of responsibility–in the presence of activation in memory of concepts related to the construct of prosociality this difference could be minimized. According to the literature, the social knowledge that is spontaneously activated in memory in the presence of the prime stimulus (stimuli that refer to the concept of prosociality) could be able to guide and shape social judgments, impressions, and intentions of the individual in a completely automatic way (Ferguson and Bargh, 2004). If so, then prime stimuli linked to the construct of altruism should prompt the subject to perceive a request for help even under conditions in which diffusion of responsibility might prevail (i.e., in the presence of other bystanders).

As said earlier, one variable that certainly influences bystander intervention is whether or not the situation is an emergency. A meta-analytic review on bystander intervention in dangerous and non-dangerous emergencies by Fischer et al. (2011) showed that the bystander effect was attenuated when situations were perceived as dangerous (compared with non-dangerous), perpetrators were present (compared with non-present), and the costs of intervention were physical (compared with non-physical). However, the bystander effect is likely in less critical situations, such as a stranded motorist or other technical problems. The effect occurs even in everyday mishaps, such as when pencils spill to the ground or when a door needs to be answered (Fischer et al., 2011).

Our study will consider the bystander effect in a non-emergency situation.

## The study

This study examines the influence of prosocial primes on bystander apathy in an online context. Suppose the probability of an e-mail response is inverse to the total of recipients who were requested help. Could this probability be reduced if prosocial concepts were activated through priming? Although the effect of diffusion of responsibility has been confirmed in impersonal settings, such as telematic communication (Blair et al., 2005), no research has focused on priming.

*Hypothesis 1.* Replies to an e-mail request for help will be reduced as the number of mail recipients increases.

*Hypothesis 2:* When the request for help is preceded by prosocial priming, no difference emerges (i.e., the number of recipients does not impact the likelihood of their response).

## Materials and methods

### Participants

One hundred eighty (110 women, 70; men; mean age 24 years, s.d 1.25) students registered in graduate courses at Palermo University were involved in the experiment. Students were contacted with a plea for help through the e-mail address book in the university databases.

### Design and procedure

A confederate who claims to be a student sent participants an e-mail message in which she was requesting help for her final thesis. The participants were randomly assigned to one of four conditions in a 2 (Bystander: 0 vs. 14) × 2 (Priming: present vs. absent) design. The confederate was a student named Paola.

#### Independent variables

##### Bystander (0 vs. 14)

The number of recipients typed in the appropriate field (i.e., To:) was manipulated so that participants would believe that 0 or 14 others had received the identical message. The participant’s name assigned to the present bystander condition was always the seventh, and others’ names were fictitious. Participants who received the mail could see that there were thirteen other recipients in the “To” field. Conversely, the names assigned to the absent bystander condition appeared to be alone.

##### Priming (present vs. absent)

In the priming condition, the plea for help was inserted within a message containing several hints of prosocial concepts. Further, an image was placed at the head of the page before the message’s text. This image recalled the idea of prosociality. The image and words used in the message have been selected through a preliminary pilot study. The message e-mail was the following:

Hello, my name is Paola Roccato. I am an ex-colleague of yours from the University of Palermo. Last year I was also attending the Psychology degree course. But for family reasons, I had to change city, and now I am in Turin, where I am doing my thesis on altruistic behavior.
Indeed, I have always been interested in studying altruistic and prosocial behaviors in intragroup and intergroup. Looking at how to heighten helping behaviors has always been my main target. I have arrived at the final project, and my thesis involves research on the relationship between helping behavior in young adolescents in contexts of social emergency. It seems well-established that a social crisis strengthens the sense of community, solidarity, and brotherhood. E as you know, these skills, in turn, increase the probability of intervening in emergencies through the implementation of helping behaviors. Maybe, I became passionate about studying prosociality because I hope this tendency will be more prevalent among people.
I need Professor Scaffidi’s slides used in class and available for students on her web page. The problem is that I can no longer access the professors’ material with the credentials I used last year since I no longer have access to the content as a student at the University of Palermo. Could you please send them to me? If you don’t have the slides, please go to Professor Scaffidi’s page and download them for me with your credentials?
Thank you very much,
Paola.

In not priming condition, the message directly contained the plea for help without any allusions to prosocial concepts, and the figure recalling the prosociality was absent. The message e-mail was the following:

Hello, my name is Paola Roccato. I am an ex-colleague of yours from the University of Palermo. Last year I was also attending the Psychology degree course. But for family reasons, I had to change city, and now I am in Turin, where I am doing my thesis on how obesity relates to socioeconomic status and identifying eating behavior mediators.

I need Professor Scaffidi’s slides used in class and available for students on her web page. The problem is that I can no longer access the professors’ material with the credentials I used last year since I no longer have access to the content as a student at the University of Palermo. Could you please send them to me? If you don’t have the slides, please go to Professor Scaffidi’s page and download them for me with your credentials?

Thank you very much,

Paola.

We have considered valid the answers arrived within 15 days. Given the logic of the experimental procedure, we had to renounce informed consent from the participants. On the other hand, the study presented itself as a harmless experiment.

##### Behavioral measure

The dependent variable was dichotomous: help or no help. Only replies containing the attachments requested by Paola were considered helpful replies.

### Data analysis

As we needed to examine the relationship between more than two categorical variables, we conducted a log-linear analysis. In particular, through the statistical log-linear, we tested the influence of the two independent variables (Bystander condition: None vs.14 bystanders; priming condition: Present vs. absent) on the dependent variable (Helping behavior: No help, helped). We used SPSS 26 software to run the statistical analyses.

## Results

Five of the one hundred eighty e-mails sent to students returned to the sender and were not computed for data analysis. As shown in Table 1, **58** (33.1%) of the 175 participants had responded at the end of the 15 days.

**TABLE 1:** Number of responses received under the four experimental conditions.

Number of recipients	Prime	Help		Total
		Not	Yes	
Alone	Absent	32	18	50
(Bystander absent)	Present	25	16	41
	Total	57	34	91
14 Others	Absent	35	5	40
(Bystander present)	Present	25	19	44
	Total	60	24	84
Total	Absent	67	23	90
	Present	50	35	85
	Total	117	58	175

We carried out a log-linear analysis to analyze the influence of the two independent variables (Bystander condition: none vs. 14 bystanders; priming condition: present vs. absent) on the dependent variable (Helping behavior: no help, helped).

A hierarchical, log-linear analysis revealed that the three-way association prime x bystander x help was significant, λ = –0.18, s.e. = 0.08, *p* = 0.03. The two-way association prime x help was significant, λ = 0.21, s.e. = 0.08, *p* = 0.01 and the two-way association prime x bystander was almost significant, λ = 0.16, s.e. = 0.8, *p* = 0.06. Finally, the two-way association bystander x help was not significant, λ = –0.14, s.e. = 0.08, *p* = 0.10.

Tests of partial associations revealed significant one-way goodness of fit association, showing that participants did not help at equal rates, χ2 (1, *N* = 175) = 20.28, *p* < 0.001. More participants did not help (66.9%) than help (33.1%).

**FIGURE 1:**
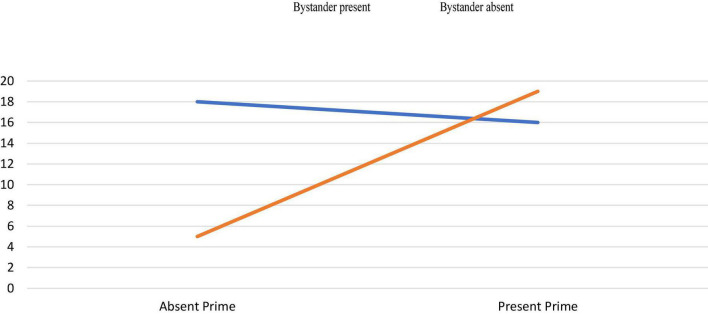
Help in each experimental condition.

A test of partial association showed that participants helped more in the prime condition (60.3%) than in the absence of prime (39.7%), χ2 (1,175) = 4.81, *p* = 0.028. This effect is significant in the present bystander condition, χ2 (1,84) = 9.66, *p* = 0.002. and it is not significant in not bystander condition, χ2 (1,91) = 0.08, *p* = 0.767. Further, a test of partial association revealed that participants in the absent prime condition helped more in the not bystander condition (78.3%) than in the 14_bystander condition (21.7%), χ2 (1,90) = 6.45, *p* = 0.011 (Figure 1).

We did not analyze the gender effect since, according to the relevant literature, the gender of the victim has not been shown to significantly influence the bystander effect in non-emergency situations or even in computer-mediated communication. Instead, gender becomes important in emergencies and dangerous circumstances, such as bullying (Latané and Nida, 1981; Eagly and Crowley, 1986; Markey, 2000; Voelpel et al., 2008; Cox and Adam, 2018; Jenkins and Nickerson, 2019; Hoxmeier et al., 2022).

## Discussion

Our research focus was dual. First, we studied the bystander effect within a virtual scenario, questioning whether the diffusion of responsibility occurs when people physically witness an event and in less traditional contexts, such as virtual settings. A second aim was to assess whether a prosocial prime in a virtual context may reduce the responsibility diffusion.

In agreement with hypothesis 1, the data suggest that the virtual presence of others decreased responses to the e-mail asking for help. As results showed, responses to an electronic plea for help lessened in the absent prime condition as the number of recipients in the mail was 14, confirming that the bystander effect occurs even in an online context. Thus, in line with Blair et al.’s (2005) research, this study showed a virtual diffusion of responsibility. From a practical point of view, studying the bystander effect in an online context might help to understand to what extent and under what conditions contacting numerous people at once in the hope of receiving a response from every person on the e-mail list is or is not a good strategy. Does this mode represent a condition that encourages the diffusion of responsibility? To what extent do we pay attention to the e-mail when we glance at other addresses in the recipient’s field? And, in any case, to what extent do we consider our eventual response indeed?

Results also showed that when the request for help was preceded by prosocial priming, the number of recipients did not influence the likelihood of their response. This result confirms hypothesis 2. It could be said that results indicated the effect of priming on helping behavior, but they also show that the presence of bystanders did not weaken the prosocial priming effect.

The data certainly highlights how powerful the effect of prime is. Behavioral priming has always been an issue of debate within social psychology. Questions have often revolved around the extent to which activation of social constructs through priming influences subsequent behavior and the degree to which this effect can also occur outside of laboratory research in real-world social contexts (Bargh et al., 2012; Cameron et al., 2012; Doyen et al., 2012; Harris et al., 2013). In our opinion, the most interesting focus of our study concerns the elicitation of prosocial priming in an online context, such as e-mail. Most research on priming in an online context has been carried out mainly in marketing (Smith and Wheeldon, 2001; Misuraca et al., 2019, 2021a; Dennis et al., 2020; Tanford et al., 2020). We do not know of any study investigating the impact of priming on bystander apathy in the online context.

Last, we must point out that we focused on assessing real helping behavior. Much research investigating the effects of prime on helping behaviors has effectively operationalized the variable “helping behavior” through “intention to help. Excluding a few exceptions (e.g., Macrae and Johnston, 1998; Abbate et al., 2013; Meier et al., 2021), primary intention to help has generally been evaluated (Garcia et al., 2002; Nelson and Norton, 2005; Pichon et al., 2007; Greitemeyer, 2009). The literature in this field tells us that intentions lead to behavior and that the stronger the intentions are, the greater the likelihood of observing the corresponding actual behavior. Yet, when helping behavior has been measured merely through the participants’ will to donate to an aid organization or by requesting participants if they are willing to participate in a subsequent experiment, participants may conform to experimenters’ requests. Using behavioral measures of prosocial behavior would undoubtedly be an essential choice to analyze the processes underlying prosocial behavior more accurately.

## Limitations and future directions

We want to make a few concluding remarks about the study’s limitations. First, some might be unconvinced by how we operationalized the “help” variable. Since only responses to e-mails containing the attachments requested by Paola were considered helpful responses, we should be questioning whether the category “NO” represents an actual bystander behavior. Other recipients could have answered “NO” because they did not access their e-mail then. We can’t check unequivocally if the recipient has read the e-mail or if they haven’t checked their e-mail in those 2 weeks. We can claim that, in addition to removing from the final count the e-mails that had come back, the university e-mail is checked by students many times a day because it is necessary for any activity in our department. Students are “induced” to continuously interact with faculty for lecture or tutorial activities or thesis or, again, for internships or, finally, for exams. We gave 2 weeks. It is unlikely that a student has not checked their e-mail. But, even so, we recognize that this can be a point of weakness.

Secondly, a weakness of studying social priming in this setting is that one cannot be sure participants fully experienced the prime. An additional would-be limitation might be the small sample size. There were 180 students in the social psychology course the year the study was done, and since the plea for help was related to the slides, it would not have made sense to contact students from other courses. Further, examining how our results are generalizable to non-students, kinds of needs, and settings is crucial.

Finally, the following studies should investigate whether the period between the time an e-mail is sent and the time it is seen influences the likelihood of help and whether the numbers of participants included in the recipient list modulate this effect. Face-to-face studies have shown that people who arrive late at the scene of an accident are less sensible for helping than those who come immediately (Cacioppo et al., 1986). A similar perception could occur when someone reads an e-mail that they feel is outdated. Another interesting direction for further research could be to investigate whether specific personality characteristics, such as the tendency to maximize or to make optimal decisions, influence the observed effect (Misuraca and Teuscher, 2013; Misuraca et al., 2015, 2021b; Misuraca and Fasolo, 2018).

## Data availability statement

The raw data supporting the conclusions of this article will be made available by the authors, without undue reservation.

## Ethics statement

Ethical review and approval was not required for the study on human participants in accordance with the local legislation and institutional requirements. Written informed consent for participation was not required for this study in accordance with the national legislation and the institutional requirements.

## Author contributions

CS: conceptualization, and writing – original draft preparation. RM, SM, CV, LV, and MR: review and editing. All authors read and agreed to the published version of the manuscript.
